# HIV-Associated Facial Lipodystrophy: Experience of a Tertiary Referral Center With Fat and Dermis-Fat Compound Graft Transfer

**Published:** 2016-12-01

**Authors:** Francisco Martins de Carvalho, Diogo Casal, Joaquim Bexiga, Juliana Sousa, João Martins, Eugénio Teófilo, Fernando Maltez, Isabel Germano, José Videira e Castro

**Affiliations:** ^a^Plastic, Reconstructive and Aesthetic Surgery Department and Burn Unit, Centro Hospitalar de Lisboa Central, Hospital de São José, Lisbon, Portugal; ^b^Hospital de Santo António dos Capuchos, Immunodeficiency Clinics, Centro Hospitalar de Lisboa Central, Lisbon, Portugal; ^c^Hospital Curry Cabral, Immunodeficiency Clinics, Centro Hospitalar de Lisboa Central, Lisbon, Portugal; ^d^Hospital de São José, Immunodeficiency Clinics, Centro Hospitalar de Lisboa Central, Lisbon, Portugal

**Keywords:** HIV, AIDS, lipodystrophy, surgery, fat graft

## Abstract

**Objectives:** HIV-associated lipodystrophy is a common comorbidity in HIV-infected patients, having a profound impact on every aspect of patients’ lives, particularly when involving the face. Hence, it is of the utmost importance to evaluate the result of any potential therapies that may help solve HIV-associated facial lipodystrophy. The aim of this article was to evaluate the outcome of patients undergoing facial lipodystrophy correction surgery within our institution. **Methods:** A retrospective analysis of the clinical charts and iconographic information of patients regarding demographics, morphologic changes, surgical option, postoperative complications, results, and patient satisfaction assessed by a 1- to 10-point scale and by the Assessment of Body Change and Distress questionnaire. **Results:** Twenty-three patients were operated on from March 2011 to April 2015. Seventy-five percent of cases were treated with fat graft injection, whereas dermis-fat grafts were applied in 25% of patients. The former had their fat harvested more commonly from the abdomen, whereas in the latter case, the graft was harvested mostly from the inner aspect of arms. The mean volume of fat injected on each side of the face was 28.5 ± 22.7 mL. On a scale from 1 to 10, mean patient satisfaction was 7.7 ± 2.8. The Assessment of Body Change and Distress questionnaire revealed statistically significant improvements. Complications occurred in 25% of cases, the most frequent being significant reabsorption. No major complications occurred. **Conclusions:** Treatment of HIV-associated facial lipodystrophy with autologous fat or dermis-fat compound graft is a safe procedure with long-lasting results and unquestionable aesthetic and social benefits.

First described in 1998,^1^ HIV-associated lipodystrophy is a common comorbidity characterized by morphologic changes (central fat accumulation and peripheral fat atrophy) and metabolic changes (hyperlipidemia and insulin resistance).^2^

According to Cabrero et al,^3^ based on a series of 965 HIV-infected patients, the prevalence of perceived body physical changes in HIV-infected patients treated with highly active antiretroviral therapy is around 55%, with the majority complaining about lipoatrophy (47%). Other authors, with smaller series, have reported rates of lipoatrophy ranging from 13% to 63%.^4^

The most common site for lipoatrophy is the face,^5^ a place not hidden by clothes. This change makes patients look ill or older, although their immune status may be excellent. This may lead to poorer adherence and even failure of antiretroviral therapy,^6^ as well as erosion of self-image and self-esteem, problems in social and sexual relations, forced HIV/AIDS disclosure, and demoralization and depression.^7^

Although objective evaluation of different treatments of facial lipodystrophy is therefore very important, few studies have been conducted concerning this subject. The purpose of this article was to evaluate the outcome of patients undergoing facial lipodystrophy correction surgery within our institution.

## METHODS

A retrospective analysis of the clinical charts and iconographic information of patients regarding demographics, morphologic changes, surgical option, postoperative complications, results, and patient satisfaction was carried out on all HIV-infected patients operated on within our institution from March 2011 to April 2015. Patient permission for taking and including photographs in the clinical charts and for publishing purposes was obtained.

### Surgical technique

Patients were evaluated for the availability of fat tissue. Those with insufficient fat tissue underwent a dermis-fat compound graft transfer, and the remaining a fat graft transfer. Patients were operated on under general anesthesia in an outpatient basis, except for the first 2 patients. They received preoperatively prophylactic antibiotic therapy with amoxicillin/clavulanic acid and metronidazole, which was continued for 5 days postoperatively.

When fat graft transfer was chosen, the Coleman technique was used, and both the donor and receptor sites were infiltrated with Klein's solution. Beside the Bichat fat pad, we focused on grafting not only the superficial nasolabial, middle, and medial cheek fat but also the deeper planes—the deep medial cheek fat compartment and *Ristow's space*. We deliberately aimed at slightly overcorrecting the fat defect.

When dermis-fat grafts were chosen, an ellipsoid-shaped dermis-fat graft measuring about 4 × 10 cm with 1-cm thickness was harvested from the inner aspect of arms, lower abdomen, medial aspect of thighs, and groin region and the primary defect primarily closed. The graft was inserted through two 5-mm-long incisions placed according to the Langer lines in or parallel to the nasolabial fold, with the dermis facing the deep aspect of the wound. The graft was secured with subcuticular interrupted 4/0 Monocryl sutures.

Patients wore a facial bandage and were instructed to avoid facial gesticulation and to keep the head elevated for 1 week.

### Patient satisfaction

Patient satisfaction was regularly assessed on a scale of 1 to 10 and by the Assessment of Body Change and Distress (ABCD) original questionnaire, both after the sixth month of follow-up.

### Statistical analysis

The data were inserted in an Excel database. Qualitative variables were expressed as percentages. Quantitative variables were expressed as means ± standard deviation. The SPSS 21.0 software was used for descriptive and inferential statistical analyses. The Kolmogorov-Smirnov test was used to assess whether variables were normally distributed. Analysis of variance and the Student *t* test were used to compare averages in normally distributed data. The Kruskal-Wallis and Mann-Whitney *U* tests were used to compare means in non-normally distributed data. Proportions were analyzed with the χ^2^ test or the Fisher exact test. Dichotomous variables were compared with the binomial test. A 2-tailed *P* value of less than .05 was considered to be statistically significant.

## RESULTS

**Figure 1:**
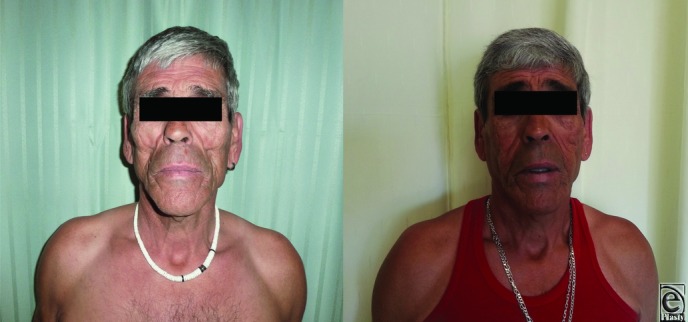
Pre- and postoperatively at 1 year: front.

**Figure 2:**
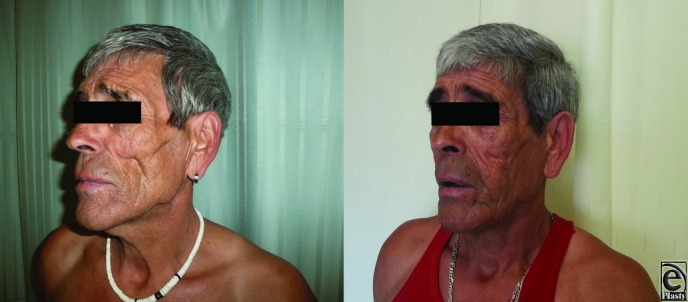
Pre- and postoperatively at 1 year: side.

Patient characteristics, technical considerations, complications, and results concerning satisfaction with the procedure are presented in Table 1 and Figures 1 and 2.

Twenty-three patients ranging in age from 35 to 67 years (50.2 ± 8.1 years) were operated on within our institution from March 2011 to April 2015. Minimum follow-up time was 6 months (average time of follow-up was 28.5 ± 12.7 months). Surgery was most commonly performed on males than on females (79.2% vs 20.8% of cases; *P* = .007).

Seventy-five percent of cases were treated with fat graft injection, whereas dermis-fat compound grafts were applied in 25% of patients (*P* = .023). In patients undergoing fat grafting, the donor site was the abdomen, dorsal cervical fat pad, abdomen and thighs, and adipomastia in 77.8%, 11.1%, 5.6%, and 5.6% of the cases, respectively. In patients undergoing dermis-fat compound graft, the graft was harvested from the inner aspect of arms, lower abdomen, inner aspect of thighs, and groin region. The first corresponded to 50% of cases of compound graft harvesting, whereas the remaining regions were used in 16.7% of cases each.

The mean volume of fat injected on each side of the face was 28.5 ± 22.7 mL, with a range from 5 to 100 mL. There was no statistically significant difference between the mean volume injected in each gender.

Three-fourths of patients (n = 17) had no complication of the volume enhancement procedure. Complications occurred in 25% of cases (n = 6), the most frequent being significant reabsorption (n = 4; 16.7%), followed by hypertrophy and hematoma (n = 1; 4.2% each). In the patients in whom significant reabsorption was as issue, progressive loss of volume up to the sixth month postoperatively was observed. One patient with significant reabsorption was reoperated on, whereas the remaining cases with significant reabsorption or hypertrophy refused reoperation. Hematoma was managed conservatively, achieving a good result.

Early on in our experience, we faced a case of hypertrophy in a patient who had his fat harvested from adipomastia. From then on, based on this experience and other reports,^8^ we stopped harvesting fat from overly hypertrophic regions. Apart from these adverse cosmetic outcomes and 1 hematoma case, we had no major complications or iatrogeny.

No differences in complication rates were found between fat graft and dermis-fat compound graft, nor between genders.

Concerning patient satisfaction, on a scale from 1 to 10, the average final result was 7.7 ± 2.8. No statistically significant differences were found between genders, nor between fat graft versus dermis-fat compound graft. Although mean patient satisfaction was only slightly different, results with dermis-fat compound grafts were, in our opinion, inferior to those of fat grafts.

The ABCD questionnaire revealed a mean increase in the score for question 7 from 2.1 ± 1.0 to 4.7 ± 0.5 (*P* < .001) and a mean decrease in the score for question 8 from 46.3 ± 8.9 to 29.6 ± 12.7 (*P* < .001).

## DISCUSSION

Facial lipoatrophy is arguably the most stigmatizing condition of HIV lipodystrophy. However, scarce data are available concerning the efficacy of different treatments for this condition. In our country, for example, no study has ever been published on this subject, as far as we could determine.

As Guaraldi et al^9^ stated, the greatest pharmacological culprits behind lipoatrophy are undoubtedly thymidine analogues, in particular stavudine and zidovudine, used for protracted periods of time, which, via mitochondrial toxicity, promote adipose tissue loss.^10^ However, other risk factors are strongly associated with lipoatrophy, such as advancing age, any use of stavudine, use of indinavir for longer than 2 years, body mass index loss, and varying degrees of duration and severity of HIV disease.^11^ New drug regimens have a lesser tendency to cause facial lipoatrophy.^12^^,^^13^ However, once settled, this condition has to be addressed surgically. The most common options include autologous fat transfer, dermis-fat compound graft transfer, and synthetic fillers.

Yang et al,^14^ using computed tomographic scans, found differences in subcutaneous cheek and Bichat fat pad fat volume up to 15 and 4 mL, respectively, per side in moderate to severe lipoatrophy versus controls. Although previously unclear, it is now well documented that the Bichat fat pad is significantly depleted, although the extent of depletion (with an average volume of 60% compared with controls) appears less than that of the superficial fat depletion (with an average volume of 45% compared with controls).^14^

Synthetic fillers can provide a good cosmetic result. However, biodegradable fillers may require multiple treatment sessions to achieve a satisfactory result and they are not long-lasting. Hyaluronic acid gel lasts for a maximum of 8 months. Poly-l-lactic acid (PLA) and calcium hydroxyapatite (CaHA) are also degraded, but the extent of the depletion may be less because the fibrosis they induce is permanent. Nonbiodegradable fillers may achieve a final and permanent result in only 1 session. However, they tend to migrate. In our series, we first removed Bio-Alcamid (polyalkylimide) gel in 3 patients because of inferior migration. Granulomas are not uncommon, especially with silicone gel and with gels containing microparticles such as polymethylmethacrylate, PLA, and CaHA. Another problem with almost all fillers is that they are expensive.^5^^,^^9^

Since the work of Coleman,^15^ it is known that lipostructure represents a safe and long-lasting method of recontouring the face with autologous tissue. Notwithstanding, its applicability to the HIV-associated facial lipodystrophy has been a subject of debate.

On the basis of the published data of Rohrich et al^16^^,^^17^ and Gierloff et al,^18^ who presented the fat compartments of the face and their aging changes, we focused on filling not only the nasolabial, middle, and medial cheek fat but also the deeper planes—the deep medial cheek fat compartment and *Ristow's space*.

With patient satisfaction as the main goal, we not only assessed it with a 1- to 10-point scale but also applied the ABCD questionnaire. This questionnaire was developed by the Adult AIDS Clinical Trials Group of the US National Institute of Allergy and Infectious Diseases and has been specifically designed for the HIV-infected population.^19^^,^^20^ The first question, the so-called question 7 (ABCD7), is about body image satisfaction and the score ranges from 1 to 5, with 5 being “very satisfied”. The remaining 20 questions are collectively called question 8 (ABCD8). This set of questions explores body change interference with habits, attitudes, and social life, and the total score ranges from 20 to 100. The higher the score, the higher the interference. Although we found it a good tool for whole-body evaluation, it is not face-oriented, which can cause some bias.

On a scale from 1 to 10, the average final result was 7.7 ± 2.8. No statistically significant differences were found between genders, nor between fat graft versus dermis-fat compound graft. Comparing the results of the ABCD questionnaire pre- and postoperatively regarding questions 7 and 8, there was an increase in mean values in question 7 and a decrease in mean values in question 8, which were statistically significant (*P* < .001). These changes suggest that there was a clear beneficial effect of the treatment used in this series.

As stated earlier, although mean patient satisfaction was only slightly different, results with dermis-fat compound grafts were, in our opinion, inferior to those of fat grafts. Worse preoperative condition, difficulty in overcorrecting, and a tendency toward reabsorption of the dermis-fat compound graft beyond the sixth month may have contributed to a perceived worse aesthetic result in the former group of patients.

In the present series, mean fat volume injected was 28.5 ± 22.7 mL per side of the face. Homologous values in the literature are highly variable and frequently include the volume injected not only in the cheek, as we did in the present article, but also in the temporal and preauricular area. Specifically addressing mean cheek volume, they range from 7.8 to 10.5 mL of fat.^21^^,^^22^ This discrepancy may be explained by a greater severity of lipoatrophy in the patients referred to our center. However, further studies, preferably performed prospectively, are warranted.

There were 3 cases of significant reabsorption. As described in the “Results” section, we noticed a general tendency of gradual reabsorption up to the sixth month postoperatively. Other authors have reported reabsorption up to 1 year, with the majority occurring 6 to 9 months after treatment, whereas others have reported any trend toward reabsorption for a minimum of 1 year.^23^^,^^24^ Interpretation of results may be confounded by the perpetuation of the same drug, host, and disease risk factors for lipodystrophy that keep affecting the native and probably the grafted fat.

A very significant advantage of autologous reconstruction of the face in patients with HIV-associated facial lipodystrophy is probably a better compliance with antiretroviral therapy.^25^ By having this opportunity at hand, patients do not have to choose between optimal medical therapy and maintenance of their original look.

Overall, surgical treatment of HIV-associated facial lipodystrophy with autologous fat or dermis-fat compound graft was a safe procedure with long-lasting results and unquestionable aesthetic and social benefits in the present series.

## Figures and Tables

**Table 1:** Summary of patients submitted to facial lipodystrophy treatment*

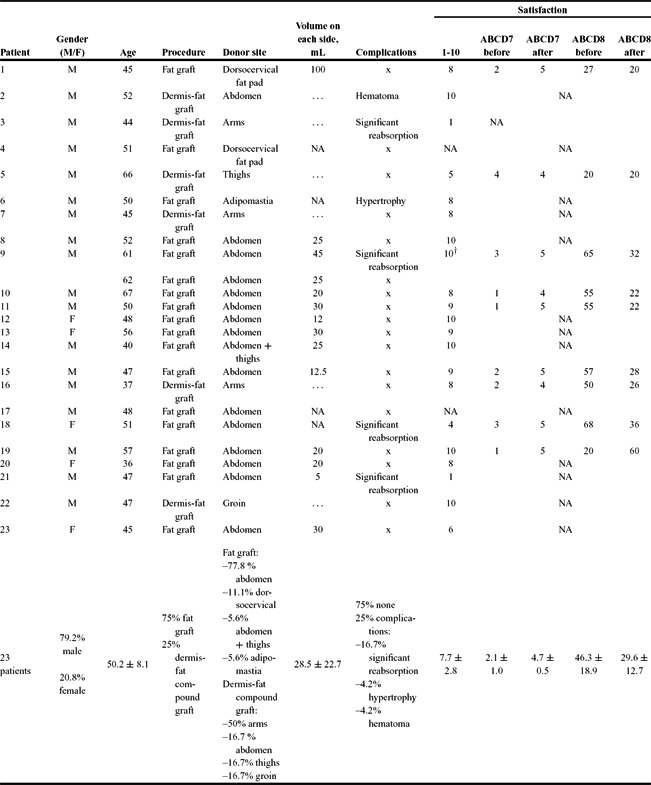

*Numeric variables are expressed as mean values ± standard deviation. ABCD indicates Assessment of Body Change and Distress; NA, not available.

†After the second procedure.
